# Ships' Surgeons: A Holiday or a Profession

**Published:** 1913-09-06

**Authors:** 


### SHIPS' SURGEONS: A HOLIDAY OR A PROFESSION.

At some time or other, usually for the sake of a change after the final examination has been passed, the qualified medical man wishes to take a sea-trip and to use his technical knowledge as a means of securing a free passage. The position at the present time may be summed up as follows. On the larger steamship lines the pay is usually from £10 a month upwards, and there is generally an arrangement whereby the ship's surgeon may or may not charge small fees to first and second class passengers. Where he is allowed to do so there is often a stipulation that fees may be charged only for attendance necessitated by an illness which had its cause before the voyage began. Such fees in any case are often subject to the approval of the commander. On cargo boats where a ship's surgeon is carried, and where there are only a few, if any, passengers, the pay may be as low as £7 per month, but on some lines a bonus of £5 a month in lieu of fees is given, which sum presumably is based upon the average income from this source which a ship's surgeon may expect, though in smaller boats it must be very inconsiderable. In the more important lines, the Cunard for instance, the pay varies from £12 15s. to £17 15s. per month, according to the size of the vessel. But on these lines a uniform must be bought; it is never, we understand, fully provided by the company, though the buttons, badges, etc., are usually given. A uniform means more than one suit, and its cost, in the case of the larger lines, may easily swallow up a month's salary. On the whole but little difficulty is experienced by the companies in filling these posts, except during the summer months. Some complain of a difficulty in finding suitable candidates, which may be interpreted to mean that a high qualification is an advantage. Except by the larger lines a doctor is often required to provide a case of instruments, though this sometimes signifies little more than a couple of clinical thermometers. The question of fees depends also on special circumstances. Thus the Royal Mail Steam Packet Company, which does not provide a uniform, pays its ships' surgeons £14 10s. a month and allows vaccination fees. It also grants an additional £2 10s. a month to ships' doctors who can speak Spanish. Again, the American Line which, flying the United States flag, employs medical men only who have an American qualification, requires a particularly strict examination of intending emigrants to the United States, since the American authorities, if unsatisfied with any emigrant, have the right to order his return to the country from which he came at the cost of the ship's company which brought him. In ships which make a round voyage the same medical man is generally engaged for the out and home journeys. Occasionally a difficulty occurs. Thus recently a case was reported to us in which a medically qualified woman passenger, travelling in a ship for which no suitable medical man could be secured, offered her services as ship's surgeon and was accepted only to be cast off from this post at a distant port where a male doctor presented himself. This, we hope, may be but the proverbial exception.

It is also possible to follow the life of a ship's surgeon as a profession. The larger lines in some cases have not merely a fleet of ships, but a "fleet surgeon," to which post a man may attain by promotion after service. The Booth Line is an example. This places its entire "medical service" under the charge of a whole-time medical superintendent. The pay is £15 a month in its cargo boats with 10s. a day shore pay, and £17 10s. a month on the passenger steamers with 11s. 8d. a day shore pay. The senior surgeon of the line receives £19 a month and 12s. 8d. a day shore pay. On the majority of this company's vessels a trained nurse is carried and in the larger ships a Royal Army Medical Corps orderly as well. On application to the various companies' offices surgeons' application forms are obtainable, and the request for them is likely to be welcomed from suitable candidates. The application forms have to be accompanied by copies generally of two testimonials. One from a clergyman is often preferred.

